# Cystoid macular edema secondary to gyrate atrophy in a child treated with sub-tenon injection of triamcinolone acetonide

**Published:** 2018

**Authors:** Şahin Alparslan, Mehmet Türkcü Fatih, Şahin Muhammed, Yıldırım Adnan

**Affiliations:** * Department of Ophthalmology, Batman Private Hospital, Batman, Turkey; ** Department of Ophthalmology, Batman Zilan Hospital, Batman, Turkey; *** Department of Ophthalmology, Dicle University, School of Medicine, Diyarbakır, Turkey; **** Department of Ophthalmology, Private Yeni Sevgi Hospital, Hatay, Turkey

**Keywords:** gyrate atrophy, triamcinolone acetonide, macular edema

## Abstract

**Purpose:** Gyrate atrophy (GA) of the fundus is a rare, progressive metabolic disease secondary to the deficiency of the pyridoxal phosphate-dependent enzyme, ornithine aminotransferase. GA may lead to cystoid macular edema (CME) resulting from chronic inflammation. We aimed to report a child case with CME secondary to gyrate atrophy.

**Methods:** Herein we presented a GA case treated with posterior sub-Tenon triamcinolone acetonide injection.

**Results:** Optical coherence tomography examination revealed the disappearance of the macular edema that is a vision-threatening complication in GA.

**Conclusion:** The present case showed that the posterior sub-Tenon injection of long acting steroids might be a promising treatment in CME secondary to GA.

## Introduction

Gyrate atrophy (GA) of the fundus is a rare, progressive metabolic disorder secondary to the deficiency of the pyridoxal phosphate-dependent enzyme, ornithine aminotransferase (OAT) [**1**]. Deficiency in OAT leads to 10 to 20-fold higher plasma ornithine levels than those of controls [**2**]. The sharply demarcated, round shaped chorioretinal atrophy with pigmented margins in the peripheral retina is the pathognomonic finding of GA. The patients with GA complain with visual deterioration and loss of night vision due to retinal atrophy, cataract formation, or cystoid macular edema (CME). At the present time, the optical coherence tomography (OCT) imaging provides more information about macular edema in GA [**3**]. In the present study, we aimed to present a child with CME secondary to gyrate atrophy treated with injection of sub-Tenon triamcinolone acetonide (TA).

## Case Report

**Fig. 1:**
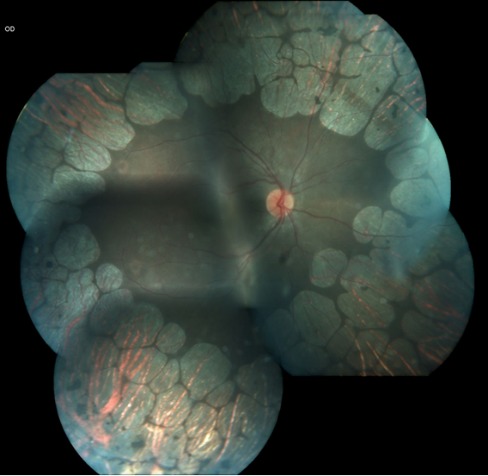
Right fundus photograph of the patient. Note the sharp demarcated lines of chorioretinal atrophy

**Fig. 2:**
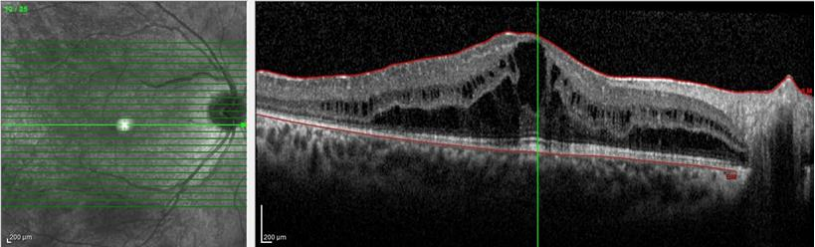
Optical coherence tomography section of the macula. There is an apparent cystoid macular edema (central foveal thickness: 625µm)

A 14-year-old female child was admitted to our retina department with a complaint of decreased vision. Her vision had deteriorated in the last few months. Her visual acuity was 2/ 10 in the right eye and 5/ 10 in the left eye. The examination of the anterior segment and measurement of the intraocular pressure were unremarkable. Fundus examination revealed typically atrophy of the retina, and the choroid (**Fig. 1**). OCT imaging showed CME, particularly in the right eye (**Fig. 2**). Serum ornithine level was 570µmol/ L (reference range 20-84µmol/ L).

The parent of the patient gave permission to the treatment only for the right eye. 40 mg/ 1cc TA was injected into the posterior sub-Tenon space.

**Fig. 3:**
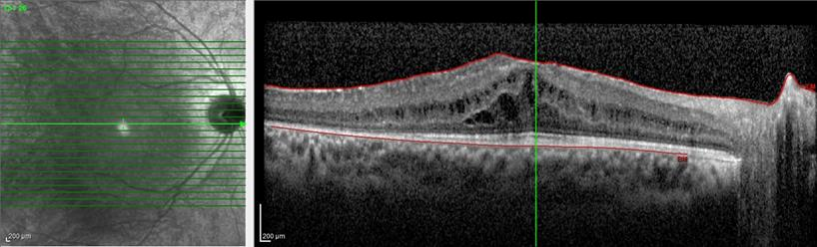
Optical coherence tomography section of the macula after triamcinolone acetonide treatment. The cystoid edema was partially recovered (central foveal thickness: 458µm)

The visual acuity in the right eye increased to 4/ 10 one month after the treatment. The intraocular pressure measurements did not show significant increase. OCT showed an apparent recovery of the CME (**Fig. 3**). The fellow eye remained stable during this time period.

## Discussion

To the best of our knowledge, this is the first case report of CME secondary to GA treated with sub-Tenon TA. We decided to treat with sub-Tenon TA injection to avoid risks of intravitreal injection such as intraocular inflammation and endophthalmitis. The recovery of the patients’ vision was promising, but the OCT showed the partial recovery of the macular edema. 

In the pathogenesis of GA, the most popular theory claims that the retina pigment epithelium pump dysfunction leads to fluid accumulation into the inner retinal layers [**4**]. High concentration of ornithine is thought to cause retina pigment epithelium toxicity [**5**]. Besides, the chronic exposure to high ornithine levels may lead to asymptomatic retinal inflammation, and macular edema may be apparent with time. The treatment should aim the ornithine-restricted diet and reduce chronic intraocular inflammation in GA patients.

Heller et al. [**6**] reported that the treatment with low protein intake and pyridoxine supplement may improve CME in GA patients. Contrary, protein restricted therapy resistant cases were reported [**7**]. Our patient was unable to administer protein restriction therapy due to economic reasons.

Steroids are routinely used in clinic practice to reduce local or systemic inflammation. Intravitreal or sub-Tenon administrations are used in ocular disorders. In our case, we preferred sub-Tenon steroid injection, because of potential complications of intravitreal injection. Previously, Vasconcelos-Santos et al. [**8**] reported a case of GA treated with intravitreal TA injection. Their patients’ visual acuity improved 20/ 80 to 20/ 50 after the treatment. Our patient showed similar improvement of visual acuity. However, CME was apparently resolved in our case. One may think that sub-Tenon injections are preferred to intravitreal injection of steroids. 

In conclusion, GA may lead CME resulting from chronic inflammation. The OCT examination revealed macular edema, a vision-threatening complication of GA. Posterior sub-Tenon injection of long acting steroids may be promising treatment in CME secondary to GA. However, further studies are needed to clarify the efficiency of steroids in GA patients with CME.

This study was presented at the 15th EURETINA Congress, 17-20 September 2015, Nice, France.

None of the authors declared a conflict interest.

The authors have no proprietary or financial interest in the products mentioned in this study, and the authors received no grants and funds in support of the study.

This paper has not been published nor submitted simultaneously for publication elsewhere.

**Compliance with ethical standards**

All procedures performed were in accordance with the ethical standards of the institutional and/ or National Research Committee and with the 1964 Helsinki Declaration and its later amendments or comparable ethical standards. An informed consent was obtained from the parent of the patient included in the study.

**Competing interests**

The authors declare that they have no competing interests.

**Funding**

None.

**Authors’ contributions**

AS and MS analyzed and interpreted the patient data. FMT and AY performed the examination of the patient. All the authors read and approved the final version of the manuscript.

**Acknowledgements**

Not applicable.
